# *Streptomyces* and their specialised metabolites for phytopathogen control – comparative *in vitro* and *in planta* metabolic approaches

**DOI:** 10.3389/fpls.2023.1151912

**Published:** 2023-06-14

**Authors:** Lachlan Dow, Marta Gallart, Margaret Ramarajan, Simon R. Law, Louise F. Thatcher

**Affiliations:** ^1^ Commonwealth Scientific and Industrial Research Organisation (CSIRO) Agriculture and Food, Acton, ACT, Australia; ^2^ Commonwealth Scientific and Industrial Research Organisation (CSIRO) Microbiomes for One Systems Health Future Science Platform, Acton, ACT, Australia; ^3^ Commonwealth Scientific and Industrial Research Organisation (CSIRO) Advanced Engineering Biology Future Science Platform, Acton, ACT, Australia

**Keywords:** *Streptomyces*, biocontrol, crop protection, microbiome, specialised metabolites, Actinobacteria, natural products, biopesticide

## Abstract

In the search for new crop protection microbial biocontrol agents, isolates from the genus *Streptomyces* are commonly found with promising attributes. *Streptomyces* are natural soil dwellers and have evolved as plant symbionts producing specialised metabolites with antibiotic and antifungal activities. *Streptomyces* biocontrol strains can effectively suppress plant pathogens via direct antimicrobial activity, but also induce plant resistance through indirect biosynthetic pathways. The investigation of factors stimulating the production and release of *Streptomyces* bioactive compounds is commonly conducted *in vitro*, between *Streptomyces* sp. and a plant pathogen. However, recent research is starting to shed light on the behaviour of these biocontrol agents *in planta*, where the biotic and abiotic conditions share little similarity to those of controlled laboratory conditions. With a focus on specialised metabolites, this review details (i) the various methods by which *Streptomyces* biocontrol agents employ specialised metabolites as an additional line of defence against plant pathogens, (ii) the signals shared in the tripartite system of plant, pathogen and biocontrol agent, and (iii) an outlook on new approaches to expedite the identification and ecological understanding of these metabolites under a crop protection lens.

## Introduction: *Streptomyces*-plant interactions and their role in crop protection

Microbial biocontrol offers an attractive ‘green’ alternative to widely used synthetic chemicals in the management of plant diseases and weeds, and there is a long history of bioprospecting for novel biocontrol strains (Barratt et al., 2018; Collinge et al., 2022). A microbial biological control agent (MBCA) is defined as a microorganism – or its natural products – used to combat pests, weeds, and diseases. MBCAs can function through one or more modes of action, (Collinge et al., 2022). These include competition for nutrients and space, parasitism, host-induced resistance, or antibiosis through the action of antimicrobial specialised metabolites, hydrolytic enzymes, or phytotoxins (Köhl et al., 2019; Boro et al., 2022). Differing modes of action can be delivered through individual microbes, with varying specificity and efficacy towards individual pathogens, or alternatively, brought together in assembled microbial consortia (Mukherjee et al., 2013; Fira et al., 2018; Mukhopadhyay and Kumar, 2020; Blake et al., 2021; Boro et al., 2022). The association of multiple MBCAs leads to combined modes of action that can act synergistically, providing more effective disease control than individual microbes (Bahkali et al., 2014; Minchev et al., 2021). Recent studies highlight that the strategic combination and deployment of multiple modes of action should be considered to overcome current gaps in the design of microbial inoculants for crop protection. Identifying the ecological function and chemical diversity of microbial specialised metabolites is essential to predict synergistic interactions in biocontrol consortia and during host microbiome assembly.

Members of the *Streptomyces* genus are of great interest as microbial inoculants due to their extensive secondary metabolism, producing over 70% of the natural products currently used in medicine and agriculture (Alam et al., 2022). In the field of plant health, *Streptomyces* spp. can exhibit beneficial functional traits through the production of specialised metabolites that enhance nutrient uptake, promote plant growth, alleviate abiotic stress, induce resistance, and prevent pathogen or pest invasion and establishment through nematicidal or insecticidal activities (Barka et al., 2016; Rey and Dumas, 2017; Newitt et al., 2019; O’Sullivan et al., 2021a; Park et al., 2023). Although predominantly associated with beneficial relationships, a small group of *Streptomyces* can produce phytotoxins and show pathogenic characteristics towards plants (Rey and Dumas, 2017; Alam et al., 2022). Several products containing *Streptomyces* microbial cells, or their purified metabolites, have been commercialised as biocontrol agents (Viaene et al., 2016; Rey and Dumas, 2017; Shi et al., 2020; Collinge et al., 2022). A non-exhaustive list of *Streptomyces*-derived specialised metabolites, which have been tested on live plants with a view to controlling plant disease, is shown in **Table 1**. The development of new biological control products is based on our ability to detect them within complex systems and confirm their bioactivity using sufficiently tailored screening methods. For example, many bioactivity screens and observable phenotypes will only detect the most abundant and/or most bioactive specialised metabolites. Compounding this issue, specialised metabolites produced by MBCAs *in vitro* are often much higher in concentration than levels produced in plant associations or in soil (reviewed in Köhl et al., 2019). Thus, better screening tools are required to place microbial specialised metabolite discovery and function into an ecological context.

**Table 1:** Synthesis of well-characterised *Streptomyces* – pathogen – host interactions available in the literature.

Metabolite	*Streptomyces* producer	Host/s	Pathogen	Mode of action	Effective concentration	Approach	References
**10-(2,2-dimethyl-cyclohexyl)-6,9-dihydroxy-4,9-dimethyl-dec-2-enoic Acid Methyl Ester**	*Streptomyces hydrogenans* DH16	*Raphanus sativus*	*Alternaria brassicicola*	Inhibited spore germination	1 mg/ml (extracted compound)	*In vitro* and *in vivo* assay	Kaur et al., 2016
**2,4-Di-*tert*-butylphenol (2,4-DTBP)**	*Streptomyces* sp. UT4A49	*Solanum lycopersicum*	*Ralstonia solanacearum*	Antibiosis (unknown mode of action)	*In vitro*: TLC-isolated metabolites from cell-free extract Pot: Cell-free extract from a 1 × 10^9^ cfu/ml culture	*In vitro* and *in vivo* assay (pot experiment)	Kaari et al., 2022
**2-methylheptyl isonicotinate and Whole cells**	*Streptomyces* sp. 201	*Raphanus sativus* L, *Brassica campestris* L *Brassica oleracea* var botrytis L	*Fusarium oxysporum* f sp raphani, *F. oxysporum* f sp conglutinans,*F. verticillioides*, *F. solani, F. semitectum, Rhizoctonia solani*	Protection from wilting	50 µg/mL (compound) 3 × 10^8^ spores/mL	*In vitro* and *in vivo* assay (pot experiment)	Bordoloi et al., 2002
**Antifungalmycin N2**	*Streptomyces* sp. N	*Vitis vinifera*; *Oryza sativa*	*Colletotrichum gloeosporioides Rhizoctonia solani*	Antibiosis and ISR	>10 ug/ml	*In vitro* and *in vivo* (excised fruit)	Xu et al., 2015; Wu et al., 2019
Blasticidin-S*	*Streptomyces griseochromogenes*	*Oryza sativa*	*Pyricularia oryzae*	Inhibits protein biosynthesis	*In vitro*: < 1 mg/ml Field: 100 - 300 g AI ha^−1^	*In vitro* and *in vivo* assays	Misato et al., 1959; Huang et al., 1964
**Conprimycin °**	*Streptomyces* sp. S4-7	*Fragaria × ananassa*	*Fusarium oxysporum* f. sp. *fragariae*	Antibiosis (inhibits cell wall biosynthesis)	Not reported	*In vitro* and *in vivo*	Cha et al., 2016
**Daunomycin**	*Actinomadura roseola*	*Capsicum annuum* cv. *Hanbyul*	*Phytophthora capsica;* *Rhizoctonia solani*	Inhibited mycelial growth	10 μg/mL (*in vitro*; 50 μg/mL (*in vivo*)	*In vitro* and *in vivo*	Kim et al., 2000
**Filipin III**	*Streptomyces miharaensis* strain KPE62302H	*Solanum lycopersicum*	*Fusarium oxysporum* f.sp. *lycopersici*	Protection from wilt	1–10 μg/mL (*in vitro*) 10 μg/mL (*in vivo*)	*In vitro* and *in vivo* greenhouse trial	Kim et al., 2012
**Geldanamycin °**	*Streptomyces hygroscopicus* var. geldanus	*Pisum sativum* L. cv. Wando	*Rhizoctonia solani*	Antibiosis	50 µg/mL 88 µg/g of soil 4.4 x 10^6^ CFU/g of soil (day7)	Pot assay	Rothrock and Gottlieb, 1984
**Gopalamicin**	*Streptomyces hygroscopicus*, MSU-625 and MSU-616	Various species, incl. *Vitis vinifera* & *Oryza sativa*	*Plasmopara vitícola Pyricularia oryzae*	Protective and curative effects	12-16 ppm (*in vitro*) 500 ppm (*in vivo*)	*In vitro* and *in vivo* greenhouse trial	Nair et al., 1994
**Indole-3-carboxylic acid**	*Streptomyces* sp. TK-VL_333	*Sorghum bicolor*	*Fusarium oxysporum*	Reduction in wilt	150 μg/ml (*in vitro*); 400 µg/mL (*in vivo*)	*In vitro* and *in vivo* greenhouse trial	Kavitha et al., 2010
**Irumamycin**	*Streptomyces subflavus* subsp*. irumaensis* AM*-*3603.	*Cucumis sativus*; *Oryza sativa*	*Botrytis cinerea* and *Colletotrichum lagenarium; Cochliobolus miyabeanus*	Inhibited growth of filamentous fungi	0.1 to 12.5 µg/ml; 200 ppm (*in vivo*)	*In vitro* assay and *in vivo* pot assay	Omura et al., 1984
Kasugamycin*	*Streptomyces kasugaensis*	*Oryza sativa*; Multiple *Pyrus* cultivars (Shinko, 20th Century and Bartlett)	*Piricularia oryzae; Erwinia amylovora*	Inhibitor of glycoside hydrolase family 18 (GH18) chitinases	100 mg/L; 100 ppm (Kasumin 2L)	Pot test and Field trial	Umezawa et al., 1965; Adaskaveg et al., 2011; Qi et al., 2021; Slack et al., 2021
**Nigericin °**	*Streptomyces violaceusniger* YCED9	Turfgrass (species unclear)	*Gaeumannomyces graminis* 151 *Sclerotinia homeocarpa Rhizoctonia solani* P	Antibiosis (unknown mode of action)	*In vitro*: TLC-isolated metabolites from cell-free extract; *in vitro*: 4.5 × 10^8^ cfu/ml soil inoculation, metabolite then detection in soil	*In vitro* and *in vivo* pot assay	Trejo-Estrada et al., 1998a; Trejo-Estrada et al., 1998b
**Oligomycin A (As1A)**	*Streptomyces libani*	*Capsicum annuum; Cucumis sativus; Oryza sativa*	*Phytophthora capsica; Colletotrichum lagenarium; Piricularia oryzae;*		3 to 5 μg/mL	*In vitro* and *in vivo* greenhouse trial	Kim et al., 1999
**Phenylacetic acid and sodium phenylacetate**	*Streptomyces humidus* strain S5-55	*Capsicum annuum* L. cv. Hanbyul	*Phytophthora capsici*	Inhibited the growth of mycelium	50 μg/ml (*in vitro*) 1,000 μg/ml (*in vivo*)	*In vitro* and *in vivo*	Hwang et al., 2001
**Rhizostreptin**	*Streptomyces griseocarneus* Benedict (strain Di944)	*Solanum lycopersicum*	*Rhizoctonia solani* Kühn	Inhibition of spore and mycelial growth	0.5 and 2 µg/mL 10^6^ cfu/g of seeds	*In vitro* and *in vivo*	Sabaratnam and Traquair, 2015
**SPM5C-1(structure unknown, but contains lactone carbonyl units)**	*Streptomyces* sp. PM5	*Oryza sativa* L. IR50	*Pyricularia oryzae; Rhizoctonia solani*	Inhibited conidial and sclerotial germination	250 µg/mL 500 µg/mL	*In vitro* and *in vivo* greenhouse trial	Prabavathy et al., 2006
Streptomycin*	*Streptomyces griseus*	Multiple *Pyrus* (Shinko, 20th Century and Bartlett) and *Malus* (Gala) cultivars	*Erwinia amylovora*		100 mg/L 1.68 kg ha^-1^	Field trial	Leben and Keitt, 1954; Adaskaveg et al., 2011; Slack et al., 2021
**Undecylprodigiosin**	*Streptomyces lividans*	*Arabidopsis thaliana*	*Verticillium dahliae*	Interacts with fungal chromosomal DNA	25 µg/µL	Fluorescent microscopy imaging	Meschke and Schrempf, 2010; Meschke et al., 2012
Validamycin* (Validamycin A)	*Streptomyces hygroscopicus* var. limoneus	*Oryza sativa*; *Arabidopsis thaliana; Triticum aestivum*	*Rhizoctonia solani, Pseudomonas syringae*, *Botrytis cinerea*, and *Fusarium graminearum*	Antibiosis. Inhibition of toxin biosynthesis Induction of plant defence responses through SA and JA/ET signalling pathways	*In vivo* (greenhouse): 10 ug/ml Recommended field dose: 10 mg/acre	*In vitro* and *in vivo*	Iwasa et al., 1970; Bian et al., 2021

* Purified metabolites registered in commercial products under different trademarks. ° directly detected from plant or soil.

In this review, we focus on the *Streptomyces* specialised metabolites that mediate host interactions with pathogens or host associated communities. We focus on the discrepancies between *in vitro* and *in vivo* analysis of *Streptomyces* biocontrol agents. This review brings together the disciplines of biocontrol and microbial ecology to compare metabolites produced by MBCAs and the range of novel specialised metabolites found in plant microbiomes which are yet to be characterised, and how these metabolites can be exploited for plant health and post-harvest applications. Finally, we highlight some of the challenges and new approaches used for *in vivo* metabolite characterisation such as the use of biosensors, and novel culturing techniques for the discovery, deployment, and manipulation of *Streptomyces* and their specialised metabolites for plant protection.

## Exploitation of *Streptomyces* specialised metabolite potential

Recent reviews estimate that *Streptomyces* species have the potential to produce at least 150,000 more specialised metabolites than currently known (Barka et al., 2016; Donald et al., 2022; Lacey and Rutledge, 2022). This metabolic potential can be attributed to their impressive diversity of biosynthetic gene clusters (BGCs), the highest of all known bacteria (Gavriilidou et al., 2022). Biosynthetic gene clusters are defined as a group of genes located physically near or adjacent to one another in the genome, which encode the enzymes required for the synthesis of a particular specialised metabolite. The cluster may also include genes for regulation of the metabolite in question, its transport, or self-resistance (Medema et al., 2015; Kautsar et al., 2020).

*Streptomyces* populations residing in soils or associated with plants exist in a resource- and space-competitive environment. The capacity to produce diverse specialised metabolites reflects the versatility required to thrive in challenging environments (Brader et al., 2014; Viaene et al., 2016; Hoskisson and Fernández-Martínez, 2018). Furthermore, for those *Streptomyces* spp. that reside as endophytes (most of which originate from bulk and rhizosphere soils), larger repertoires of specialised metabolites may be required, both to outcompete other organisms in multiple ecological niches (e.g., soil and plant endosphere), and as signals mediating interactions with host and associated microbiome. Given the size of BGCs, the expression of the biosynthetic components and subsequent biosynthesis of the specialised metabolites are energy demanding processes. To compensate for this energy cost, the benefit of their biological activity must be substantial, and the process tightly regulated (Hoskisson and Fernández-Martínez, 2018). In addition, the bacterium must maintain physiological regulation of specialised metabolite production for survival, growth, and sporulation processes. A detailed review by Hoskisson and Fernández-Martínez (2018) reports the complex external and internal factors influencing the regulation of specialised metabolite production in *Streptomyces* and other Actinobacteria – including sensors, regulators, signalling molecules, and metabolite precursors.

Several studies have targeted the isolation of new *Streptomyces* species (spp.) and other MBCAs from plant compartments, with the aim of identifying novel microbial strains and specialised metabolites for use against plant diseases (Brader et al., 2014; Collinge et al., 2022; Wang et al., 2022). For example, endophytic *Streptomyces* isolates collected from the roots of healthy wheat plants in Western Australia suppressed wheat fungal diseases *in planta* and promoted expression of genes involved in plant defence (Coombs and Franco, 2003; Franco et al., 2007; Belt et al., 2021; O’Sullivan et al., 2021b). While metabolites were not examined *in planta*, fungal suppression was attributed to antifungal metabolites, the activity of which was detectable using *in vitro* assays and cell-free fermentation extracts. Endophyte culture collections such as these, among others (Matsumoto and Takahashi, 2017), provide a great experimental resource to study the potential plant colonisation of *Streptomyces* MBCAs across host species and genotypes, and for analysis of *in planta* metabolite production.

An underexplored component of the *Streptomyces* metabolome associated with plant interactions are its volatile organic compounds (VOCs). Often, these metabolites are studied *in vitro* using microbes grown in the absence of interacting organisms (reviewed in (Korpi et al., 2009; Meredith and Tfaily, 2022)). However, *Streptomyces* can be prolific producers of VOCs, with *in vitro* studies detecting 40 or more VOCs from individual strains (Li et al., 2010; Cordovez et al., 2015). Produced in small quantities in their ecological niches, *Streptomyces* VOCs can be exploited in post-harvest crop protection to reduce chemical residues on foods (Zhao et al., 2022). Commonly identified antifungal VOC specialised metabolites from *Streptomyces* spp. and other MBCAs include dimethyl disulfide and dimethyl trisulfide (Li et al., 2010; Kang et al., 2021). More recently β-caryophyllene, l-linalool, and 2-ethyl-5-methylpyrazine have been isolated from a range of *Streptomyces* spp. and can suppress fungal rot or spot diseases in fruits and nuts (Lyu et al., 2020; Boukaew et al., 2021; Gong et al., 2022). *Streptomyces* non-volatile specialised metabolites can also play an important role in the management of post-harvest disease in fruit. For example, the macrolides lucensomycin and 32,33-didehydroroflamycoin, or reveromycin polyketides isolated from fermentation broths of *Streptomyces* spp. have been effective in inhibiting fruit rot caused by the fungal pathogen *Botrytis cinerea* in grapes, tomatoes, and strawberries (Lyu et al., 2017; Kim et al., 2019; Kim et al., 2020; Nguyen et al., 2021). In addition, the antimicrobial polymer Epsilon-poly-L-lysine isolated from several *Streptomyces* spp. is widely used in the agricultural and pharmaceutical industries and has activity against post-harvest fungal diseases, such as grey mould (*B. cinerea*), blue mould (*Penicillium expansum*), and anthracnose (*Colletotrichum gloeosporioides*) in avocado, mango, and papaya fruits [(Bai et al., 2022); also reviewed in Wang et al. (2021)].

## Challenges of traditional *in vitro* approaches for MBCA and specialised metabolite discovery

In our effort to better understand and exploit novel MBCAs and their specialised metabolites, culturable microbes of interest are typically characterised from collections, in isolation. This involves growing individual strains on nutrient medium under controlled conditions (Collinge et al., 2022). However, changes in nutrient medium can lead to the isolation and identification of different specialised metabolites with varying efficacy against targeted pathogens (Rateb et al., 2011; Rashad et al., 2015; Hemphill et al., 2017; Köhl et al., 2019; Pan et al., 2019). The nutrient concentration in conventional culture media is often vastly different from those found in soils and plant tissues and may inadvertently disadvantage the growth and activity of specific microbes of interest (Köhl et al., 2019). Nutrient stress or alteration studies, commonly referred to as OSMAC approaches (One Strain Many Compounds, reviewed in Pan et al., 2019), shed light on the effects of nutrient limitation in activating the production of specialised metabolites in *Streptomyces* spp. (reviewed in Hoskisson and Fernández-Martínez, 2018). For example, the depletion of sugar or other nutrients is commonly associated with the activation of microbial secondary metabolite production (e.g., antibiotics), as a means of sensing microbial competition in the environment and is an important survival mechanism (Hoskisson and Fernández-Martínez, 2018; Van Bergeijk et al., 2020). Apart from nutrient depletion, factors influencing the morphology of *Streptomyces* mycelia, or its exploratory growth mode are also emerging as triggers for differential BGC expression *in vitro* versus *in planta* (Dobson et al., 2008; Celler et al., 2012; Shepherdson and Elliot, 2022).

While *in vitro* studies have been fruitful in isolating potential *Streptomyces* MBCAs and identifying some of the signals that elicit specialised metabolite production, genome sequencing has revealed that this genus encodes far greater metabolic potential than was previously thought. Furthermore, our understanding of the ecological signals that trigger BGC expression is far from complete. In fact, the metabolic potential of *Streptomyces* may be underestimated by up to 90%, with the handful of compounds detected in laboratory grown cultures being a mere snapshot of their genomic potential (Rutledge and Challis, 2015; Barka et al., 2016; Rey and Dumas, 2017; Hoskisson and Fernández-Martínez, 2018; Van Bergeijk et al., 2020). Indeed, some individual strains possess more than 100 putative BGCs (Rutledge and Challis, 2015; Rey and Dumas, 2017; Moore et al., 2023). When the biotic or abiotic conditions required to activate BGC expression are unknown, the BGC is considered ‘silent’. Others are termed ‘cryptic’ if the specialised metabolite, activity, or phenotype cannot be linked to a predicted BGC. Recently, the structural characteristics and genomic clustering of BGCs have facilitated the development of several BGC predictive genome mining tools, such as AntiSMASH (Blin et al., 2021). However, more advances are required to identify conditions inducing BGC expression, the chemical structure of the resulting metabolites, and their function.

This large, underexplored world of *Streptomyces* metabolic potential leads us to question current approaches used for bioprospecting and MBCA discovery. To date, the screening of most available MBCAs and their specialised metabolites have been conducted *in vitro*, by culturing microbe and pathogen in isolation. These experiments restrict our ability to predict the behaviour of MBCAs *in planta*. As such, *in vitro* screening methods to identify biocontrol agents have overlooked strains which, when subsequently grown *in planta*, produce positive results. Recently, Besset-Manzoni and co-workers (2019) asked the question “Does *in vitro* selection of biocontrol agents guarantee success *in planta*?” Using a library of over 200 bacteria isolated from soil, the authors screened for *in vitro* growth inhibition against the wheat fungal pathogen *Fusarium graminearum*, using dual-culture agar plate assays and cell-free supernatants containing secreted compounds from liquid cultures. Low correlation between assays was observed, suggesting growth media affected the production of antifungal compound(s) and/or co-culturing bacteria and pathogen may be required to elicit antifungal metabolite production. A subset of bacterial strains evaluated for disease suppression on wheat indicated that most active strains *in vitro* were not necessarily the most effective *in planta*, and vice versa. With a focus on screening *Streptomyces* spp. and other Actinobacteria against the wheat pathogen *Fusarium pseudograminearum*, O’Sullivan independently arrived at similar outcomes (O’Sullivan et al., 2021b). These studies highlight not only a lack of translation from *in vitro* screens to *in planta* bioactivity, but also that conclusions made solely on *in vitro* screens may lead to promising biocontrol microbes being overlooked.

## Bespoke *in vitro* approaches for specialised metabolite production

**Figure 1:**
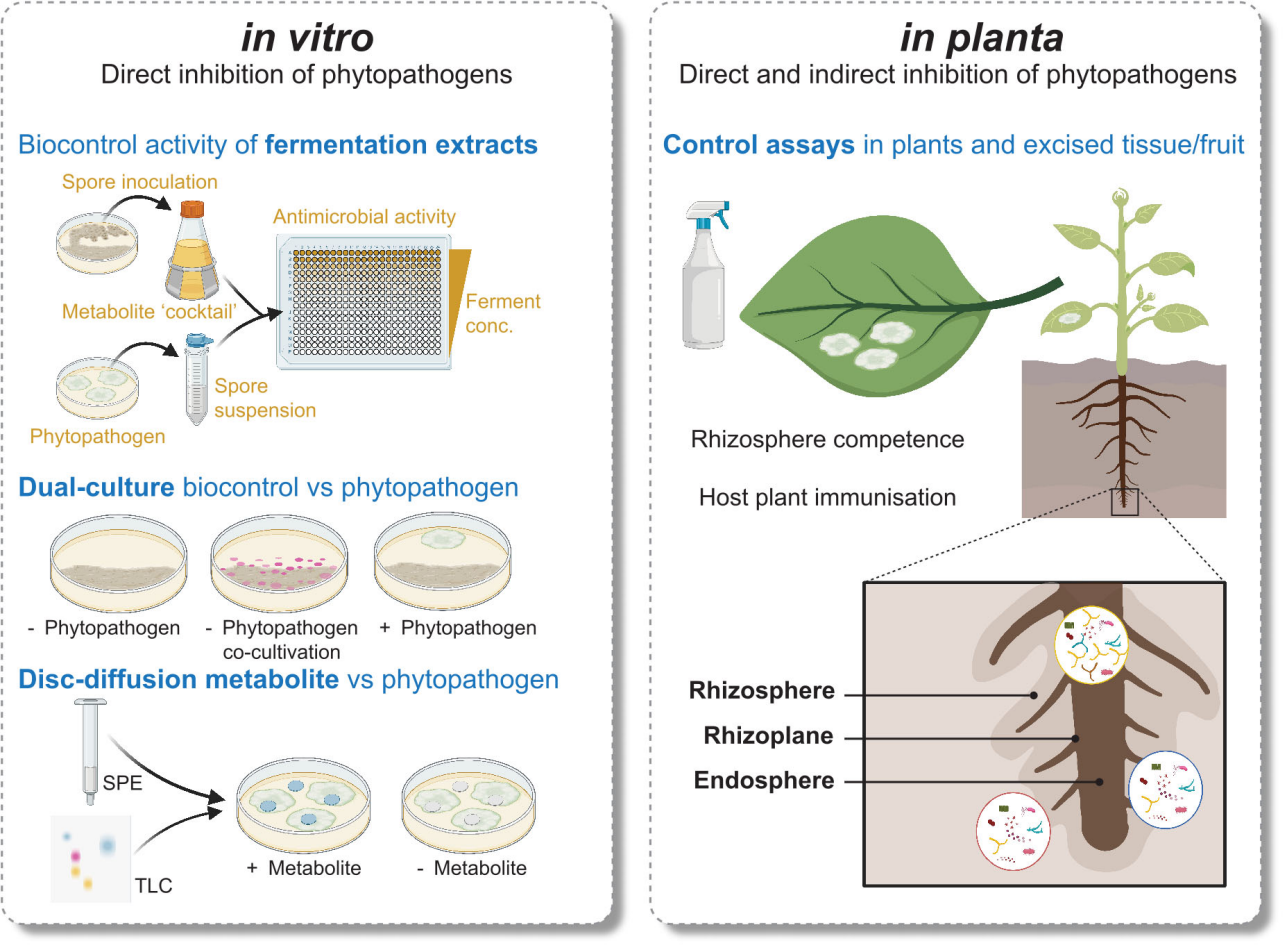
Comparison of several *in vitro* and *in planta* approaches used to discover and characterise *Streptomyces* MBCA and specialised metabolites.

While *in vitro* workflows enable isolation of specialised metabolites from pure cultures, they are only suitable for MBCA that can be cultured, and lack any insight into the behaviour of the isolate in the environment (**Figure 1**). We can apply our knowledge of the plant and microbe relationships influencing *Streptomyces* secondary metabolism to tailor *in vitro* approaches better aligned to an ecological context. For example, *Streptomyces* co-cultivation with other microbes has proven to be quite useful. In 2017 Yu and coworkers studied capacity of *Streptomyces* spp., non-*Streptomyces* bacteria, and fungi to increase biocontrol activity of *Streptomyces rimosus* against the plant pathogen *Fusarium oxysporum* f. sp. *cucumerinum* through co-culturing (Yu et al., 2017). Co-cultivation with fungal species recorded the highest induction ability, indicating perception of fungal-derived signals may be required for the activation of antifungal BGCs and their compounds. Fungal cell wall chitin subunits and their derivates also induce expression of antibiotics in *Streptomyces* (Nazari et al., 2013). Further examples of beneficial *in vitro* microbial interactions are the co-culturing of *Streptomyces coelicolor* and the soil bacterium *Myxococcus xanthus*, leading to a 20-fold increase in production of a polyketide antibiotic (Pérez et al., 2011), and the co-culturing of multiple *Streptomyces* spp. to induce antibiotic production (Ueda et al., 2000).

*Streptomyces*-derived elicitors can also be used to modulate secondary metabolism and promote expression of cryptic BGCs. For example, the linear azole-containing peptide goadsporin - originally named after its ability to stimulate sporulation - isolated from the soil *Streptomyces* sp. strain TP-A0584 promotes specialised metabolite production in a broad range, but not all, *Streptomyces* spp. (Onaka et al., 2001; Arnison et al., 2013; Onaka, 2017). In another example, Yamanaka et al. (2005) identified the siderophore desferrioxamine E produced by *Streptomyces griseus* and other members of Actinobacteria, induces aerial mycelium formation, and antibiotic and pigment production. Co-cultivation and elicitation studies provide a mechanism for activating and deciphering the inter- and intradomain interaction signals that manipulate production of *Streptomyce*s metabolites, and how these may play out in plant-associated environments.

## *Streptomyces* in the plant-soil environment

The use of next-generation sequencing technologies to study microbial diversity has substantiated long-standing hypotheses that *Streptomyces* spp. dominate microbial communities across soil ecosystems, especially in the root microbiome (Goodfellow and Williams, 1983; Viaene et al., 2016; Matsumoto and Takahashi, 2017). While the relative abundance of *Streptomyces* differs among plant species and genotypes, studies generally agree on their strong enrichment in the root endosphere (within the root), compared to the rhizosphere (soil surrounding the root) and bulk soil (reviewed by Viaene et al., 2016). For example, *Streptomyces* are enriched in the root endosphere of *Arabidopsi*s *thaliana* (Arabidopsis) and rice plants (Bulgarelli et al., 2012; Edwards et al., 2015). Furthermore, Singer et al. (2019) found a consistent enrichment of *Streptomyces* in the root endosphere and rhizoplane (the root surface) of four different switchgrass genotypes compared to rhizosphere and bulk soils.

Although edaphic factors are the main drivers of root microbiome assembly (Bulgarelli et al., 2013), several studies indicate that *Streptomyces* interactions are host-dependent (Bakker et al., 2014; Becklund et al., 2016). This suggests that host-derived signals can shape the plant-associated microbiome composition in a manner that favours *Streptomyces* diversity and richness. In line with this, Fitzpatrick et al. (2018) reported the differential capacity of angiosperm species to specifically recruit *Streptomyces* under drought-stress conditions. The high rates of *Streptomyces* colonisation under stressful environmental conditions could also be explained by the spore-forming ability of Actinobacteria, which provides greater resilience compared to less hardy bacterial lineages (Nessner Kavamura et al., 2013; Taketani et al., 2017). The production of antimicrobial compounds by *Streptomyces* that inhibit the growth of competing microbes is another leading hypothesis for their abundance in plants (Fitzpatrick et al., 2018). While the use of tagged strains and electron microscopy have further confirmed *Streptomyces*’ ability to colonise root structures, little is known about the host-selection mechanisms leading to *Streptomyces* root recruitment. For instance, studies combining DNA profiling and stable isotope labelling techniques to identify taxa that can metabolise photosynthates have demonstrated that *Streptomyces* struggle to compete for root exudates in the root microbiomes of Arabidopsis and wheat (Prudence et al., 2021; Worsley et al., 2021). Taken together, our (arguably restricted) understanding of the strategies for *Streptomyces* recruitment and the unknown factors driving root colonisation, suggests the presence of host-specific mechanisms and *Streptomyces* preference for host traits.

## *In planta* approaches for biocontrol and specialised metabolite discovery

Culture-dependent MBCA research has left many knowledge gaps: How do MBCAs behave *in planta*? Are they producing the same metabolites as observed in pure culture, and if not, what else might they be producing? *In vitro* experiments suggest that *Streptomyces* secondary metabolite biosynthesis is increased by compounds such as phytohormones (Worsley et al., 2020) and chitin (Nazari et al., 2013), indicating that *in planta* production of antimicrobial compounds is influenced by plant host and nearby microbes. As *Streptomyces* secondary metabolite diversity seems linked to interactions with hosts and microbial communities, future research on the characterisation of *Streptomyces* silent BGCs and cryptic secondary metabolites should take place in response to host environments (Seipke et al., 2012; Nazari et al., 2013).

**Figure 2:**
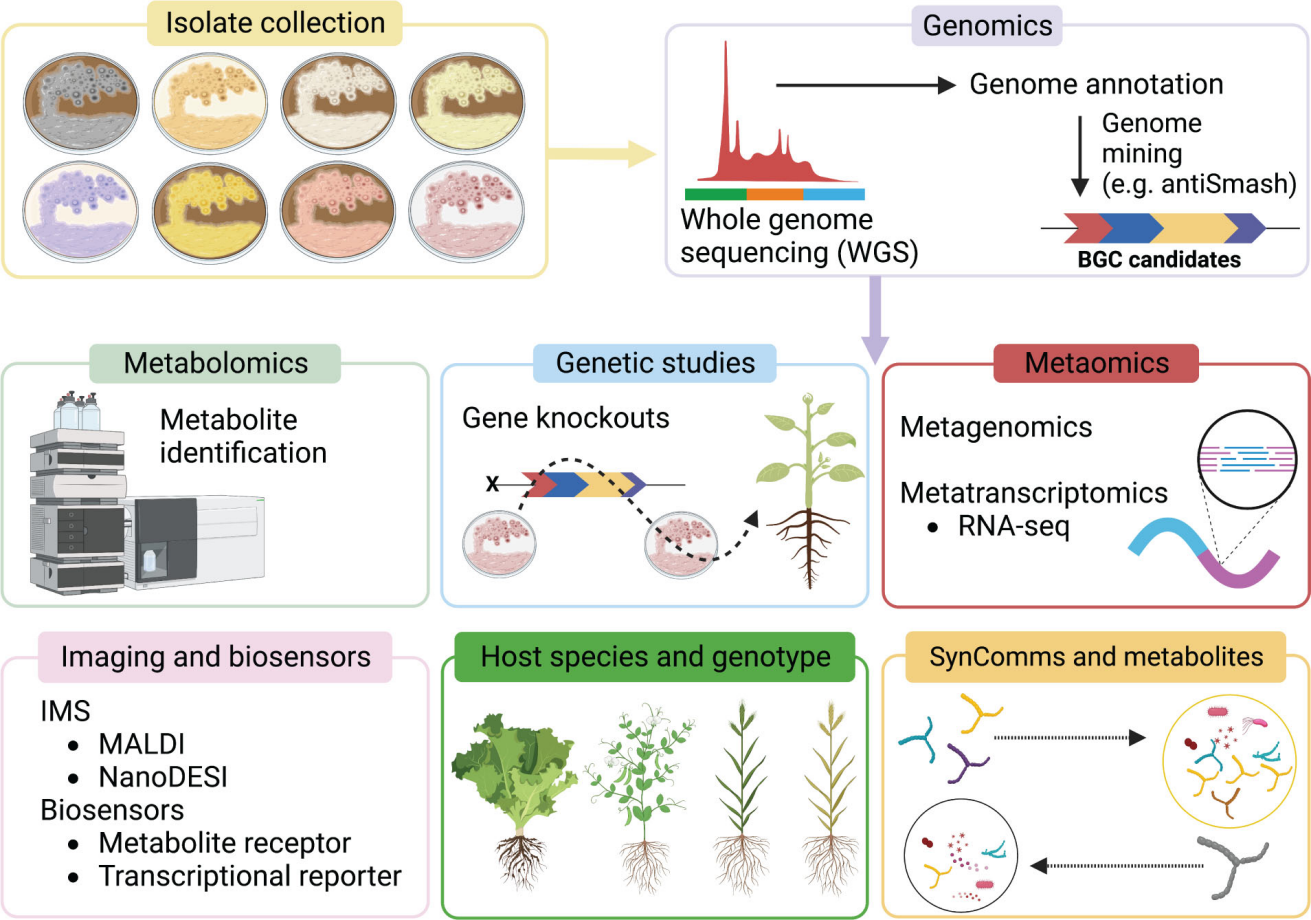
Schematic of current and emerging approaches used to identify and characterise *Streptomyces* MBCA and specialised metabolites. Tools such as genetic studies, metaomics, imaging mass spectrometry (IMS), biosensors, and new bioinformatics approaches are particularly informative components for functional characterisation.

Recent advances in metabolomics, metatranscriptomics, and metagenomics has helped to bridge the knowledge gap, shedding light on the activity of individual biocontrol agents within soils, the rhizosphere, and the endosphere, including the metabolic potential of the entire microbial community associated with plants (**Figure 2**).

### Omics approaches reveal the true metabolic potential of plant and soil microbiomes

The past two decades have witnessed remarkable advances in the speed and accuracy of Omics technologies used to quantify and characterise an organism’s complement of biopolymers (DNA, RNA, and proteins) and metabolites. Further developments have expanded the scale of these Omics approaches to the community level, capturing an unprecedented view of the taxonomic and functional diversity of microbes that populate plants and their environments (Metaomics). Metagenomic surveys have been used to great effect in cataloguing the genetic potential encoded in complex microbial communities and in the identification of putative BGCs involved in the biosynthesis of specialised metabolites (Santana-Pereira et al., 2020; Van Goethem et al., 2021; Chen et al., 2018). Metatranscriptomics and metaproteomics have enhanced these insights with global snapshots of expression dynamics, providing community-level information on the magnitude, timing, and regulatory factors associated with BGC production (Anderson and Fernando, 2021; Edlund et al., 2018). Finally, metabolomics completes this picture by identifying the totality of metabolites present in complex environmental samples, and in so doing, resolving the link between the genetic potential of microbial consortia and the specialised metabolites they produce. In this way, Metaomic approaches have identified (and begun to close) the considerable gap between predicted and experimentally verified microbial specialised metabolites. For example, two similar studies by Crits-Christoph and colleagues used a combination of metagenomic and metatranscriptomic approaches to identify several hundred novel BGCs from understudied bacterial phyla in soil samples (Crits-Christoph et al., 2018; Crits-Christoph et al., 2022). This vast diversity of BGCs in bulk soils is corroborated by the works of Carrión and colleagues, who in 2019 combined multiple metaomics techniques to quantify the number of BGCs in root microbiomes grown in disease suppressive soil (Carrión et al., 2019). Out of the 700 BGCs that were detected in this study, only 10 could be confidently assigned with a metabolite product. Further metatranscriptomic investigation indicated that none of those ten well characterised BGCs were expressed *in planta*. Instead, a handful of uncharacterised BGC were correlated with disease suppression, one of which was confirmed via isolation of the bacterium harbouring the BGC. A mutant line, with deletions within the BGC failed to suppress disease like the wild type did. Such metaomic studies clearly show that we have only begun to scratch the surface when it comes to metabolic potential in soils and plant microbiomes, however technical hurdles remain to better understand this potential. Without sufficient sequencing read depth (bot RNA and DNA sequencing) BGCs can be missed, or have very low transcript abundance. And while identifying BGCs in genome sequence data is straightforward, assigning a metabolite product nevertheless requires experimental validation.

While bulk soil contains vast numbers of BGCs, producing almost entirely unknown metabolites from extremely diverse prokaryote taxa, plant microbiomes are considerably more streamlined, with *Streptomyces* being one of the major taxa. This was recently demonstrated by Dror and colleagues, who utilised a range of metabarcoding and metagenomics techniques to compare the abundance of BGCs from the roots of lettuce, tomato, and bulk soil (Dror et al., 2020). Focusing on those BGCs encoding polyketide synthase (PKS) and non-ribosomal peptide synthase genes (NRPS), the authors showed that *Streptomyces* spp. were responsible for many of the BGCs expressed in roots. The researchers were able to assign a putative metabolite product for a larger portion of those BGCs expressed and identify the same gene cluster from other root metagenomes. Using similar metabarcoding techniques, Aleti et al. investigated potato rhizosphere samples from Peru (Aleti et al., 2017), again finding *Streptomyces* comprising a substantial portion of the rhizosphere community, while encoding over one thousand putative PKS and NRPS gene clusters. Interestingly, in both studies, some of the most abundant and highly expressed gene clusters detected were encoded by *Streptomyces* spp., and the predicted metabolite products matched characterised specialised metabolites with known antibiotic properties. With respect to plant diseases, this supports the long-held hypothesis that *Streptomyces* spp. are ‘recruited’ by plants to exploit the strong antifungal and antibacterial metabolites they produce. The relatively high success in assigning putative metabolite products to common or abundant gene clusters in plant microbiomes observed here also suggests these gene clusters represent microbial functions of particular benefit to the host plant. Overall, these studies demonstrate that complementary metaomic approaches can be used to comprehensively detect and characterise the MBGC potential of plant and soil microbiomes, which greatly increase the scale and speed of research surveying for novel pharmaceutical and agricultural products, as well as unravelling the role MBGCs have in shaping plant-microbe ecology and evolution.

### *In planta* analyses of potential MBCA specialised metabolites

In comparison to studies using sequencing-based techniques to detect the presence and/or transcription of genes encoding antifungal metabolites, there is a paucity of works in which active metabolites are measured *in planta*, or its biosynthesis confirmed via knockout mutants of the MBCA. Even by widening the focus of this review to include MBCAs of other bacterial genera, very few studies have been able to confirm whether active metabolites produced *in vitro* account for the biocontrol activity seen *in planta*. In contrast, more attention has been given to studying the other half of pathogen-MBCA interactions, through the analysis of toxins produced by fungal pathogens, such as those produced during head blight infections by *Fusarium* sp. (Palazzini et al., 2007; Palumbo et al., 2008; Palazzini et al., 2016).

Several studies have been able to assign *Streptomyces* specific metabolites to disease suppressive activity in certain soils and plant species. In 2016, Cha and colleagues investigated soil that was suppressive to *Fusarium* wilt in strawberries (Cha et al., 2016). They isolated a strain of *Streptomyces* (sp. S4-7) from this soil, and through mutagenesis and chemistry studies, assigned the antifungal activity to a thiopeptide they named conprimycin. When introduced to strawberry plants challenged with *Fusarium*, wild type S4-7 provided the plant host with an increased resistance to *Fusarium*, while conprimycin deficient mutants did not. Interestingly, mutants deficient in ectoine production (a small molecule implicated in protein chaperone interactions and osmoprotectant roles) were also conprimycin deficient, and unable to control *Fusarium* wilt (Cha et al., 2016). This work mirrors earlier studies by Rothrock and Gottlieb (1984), who asserted that the benzoquinone geldanamycin, produced by *Streptomyces* sp. in *Rhizoctonia* resistant soils, was one of the primary active metabolites in the soil (Rothrock and Gottlieb, 1984). Also focusing on *Rhizoctonia*, works by Trejo-Estrada and colleagues detected nigericin and geldanamycin from *Streptomyces* sp. YCED9 in soil and thatch grass samples (Trejo-Estrada et al., 1998a). Geldanamycin was also identified as the active metabolite produced by *Streptomyces melanosprofaciens* EF-76 in a more recent study (Agbessi et al., 2003). Geldanamycin deficient mutants of *S. melanosprofaciens* EF-76 were unable to control *Streptomyces scabies* (the causal agent of potato scab) in field trials, while the wild type displayed biocontrol activity. Collectively, these studies show that specific gene clusters can account for disease resistant soils, as well as the relative ubiquitousness of geldanamycin in these systems, and highlight the power of gene knockouts to confirm MBCA modes of action.

Similar reverse genetic approaches have been used *in planta* to identify specialised metabolites from well-studied MBCAs. A combination of culture-independent and culture-dependent methods were utilised to identify a specific BGC in *Pseudomonas* sp. CH-C52 from soils suppressive to *Rhizoctonia solani* infections. Knockouts of this gene cluster resulted in *Pseudomonas* sp. CH-C52 that were ineffective at protecting sugar beet seedlings from the disease (Mendes et al., 2011). The metabolite in question, thanamycin (a member of the syringomycin family), was later identified using innovative methods, such as mass spectrometry imaging and mass spectral networking (Watrous et al., 2012; van der Voort et al., 2015). Again, in *Pseudomonas*, Chen et al. (2018) studied the microbiome of wheat heads, isolating the novel biocontrol *Pseudomonas piscium*, which protected the host from *Fusarium graminearum* infections. Using knockout mutants, the authors identified that antifungal activity was mediated by phenazine-1-carboxamide (Chen et al., 2018). In another effort to understand the biocontrol of *F. graminearum*, Crane et al. (2013) were able to quantify the antifungal lipopeptide iturin produced *in planta* by the commercial MBCA strain *Bacillus amyloliquefaciens* “TrigoCor”. While the population of *B. amyloliquefaciens* remained constant during the experimental period, the level of iturin on wheat heads decreased over time. This study perfectly illustrates the need to adopt analytical techniques within *in planta* studies, to confirm that the desired metabolites of MBCAs are present when and where required.

The research summarised above, discussing both untargeted analyses of microbiome secondary metabolism and focused studies on single bacteria, highlights the dichotomy currently present in biocontrol research. While plant microbiomes present a nearly inexhaustible wealth of novel metabolites that can be exploited to combat plant pathogens, MBCAs are typically first identified via *in vitro* methods, with little *in planta* analyses to understand their behaviour once the host plant has been colonised. These *in vitro* tests rely on the active metabolite(s) being synthesised *in planta*, an environment far removed from that of an agar plate, which means MBCA screenings are biased towards isolates that express gene clusters in both settings. Detecting MBCA-derived metabolites *in planta* is rarely conducted but is necessary to validate many assumptions within the field of research. While analytical chemistry techniques may not be sensitive enough to detect the metabolites in question, biosensor-based methods have the potential to detect low abundance metabolites *in planta*, and even provide spatial information.

### Biosensors as direct or indirect reporters of MBCA and specialised metabolite activity

Biosensors are enzymes, antibodies, or microorganisms, which can detect specific molecules in a sample and report their presence as a quantifiable signal. As such, they can be engineered to respond to specialised metabolite activity (Liu et al., 2015). For example, under the control of a metabolically responsive promoter or a specific metabolite ligand-binding receptor, biosensors are often expressed in a microbial host that in the presence or absence of a target molecule in the microbes environment activates a signal, such as fluorescence. Biosensors with sufficient sensitivity and/or adequate abundance of the reporter protein can facilitate spatial and temporal detection of metabolites. Biosensors have been frequently applied to *Streptomyces* within *in vitro* settings to identify conditions that improve antibiotic production or in molecular approaches to manipulate BGC expression (Ostash et al., 2010; Guo et al., 2015; Sun et al., 2017; Kasey et al., 2018; Li et al., 2019). However, they are also useful tools to gain an understanding of microbial metabolites in ecological settings (Leveau and Lindow, 2002; Zhang and Keasling, 2011). For example, Hansen et al. (2021) developed a biosensor to detect the bacterial specialised metabolite 2,4-diacetylphloroglucinol (DAPG), involved in phytopathogen suppression, in a variety of grassland soils. Subsequently, they used the biosensor platform to investigate how interactions within the *Pseudomonas* genus specifically affected the production of DAPG and induced or suppressed plant-beneficial activities in other rhizobacteria (Hansen et al., 2022). Similarly, Masarra et al. (2015) used a *Chromobacterium violaceum* mutant that produces the pigment violacein in response to quorum sensing molecules as a ‘native’ biosensor to screen a library of 63 soil-isolated *Streptomyces* isolates for metabolites with quorum quenching activity. Finally, Chane et al. (2019) developed a biosensor to monitor quorum signal molecule detection or degradation in the Actinobacteria biocontrol *Rhodococcus erythropolis* in response to co-inoculation onto potato tubers with the soft rot pathogen *Pectobacterium atrosepticum*.

Biosensors can also be incorporated within a plant host to report on host detection of a MBCA or its metabolites. For example, in a study by Vergnes et al. (2020) a GUS reporter under the control of the Arabidopsis Pathogenesis-related1 (PR1) promoter was used to screen for *Streptomyces* species that could stimulate defence signalling pathways in transgenic Arabidopsis carrying the reporter. They found one *Streptomyces* strain (AgN23) that triggered jasmonic acid and salicylic acid dependent responses. In a separate study, Belt et al. (2021) described the use of a luciferase reporter under the control of the stress-responsive Glutathione S-transferase-7 (GSTF7) promoter, to screen a collection of Actinobacteria to identify candidates that could activate plant stress-responses. In this study, a *Streptomyces* strain (KB001) and its fermentation extract were identified, which helped protect Arabidopsis plants against two fungal pathogens, *Sclerotinia sclerotiorum* and *Rhizoctonia solani*. Similarly, a *PR1:GUS* gene reporter was used to verify the activation of salicylic acid signalling pathway in transgenic Arabidopsis plants using fermentation broth of *Streptomyces* strain JCK-6131 (Le et al., 2021). These studies reinforce the importance of studying MBCAs in a tripartite system, where there efficacy involves priming plant immunity, which is overlooked in simple *in vitro* systems.

**Figure 3:**
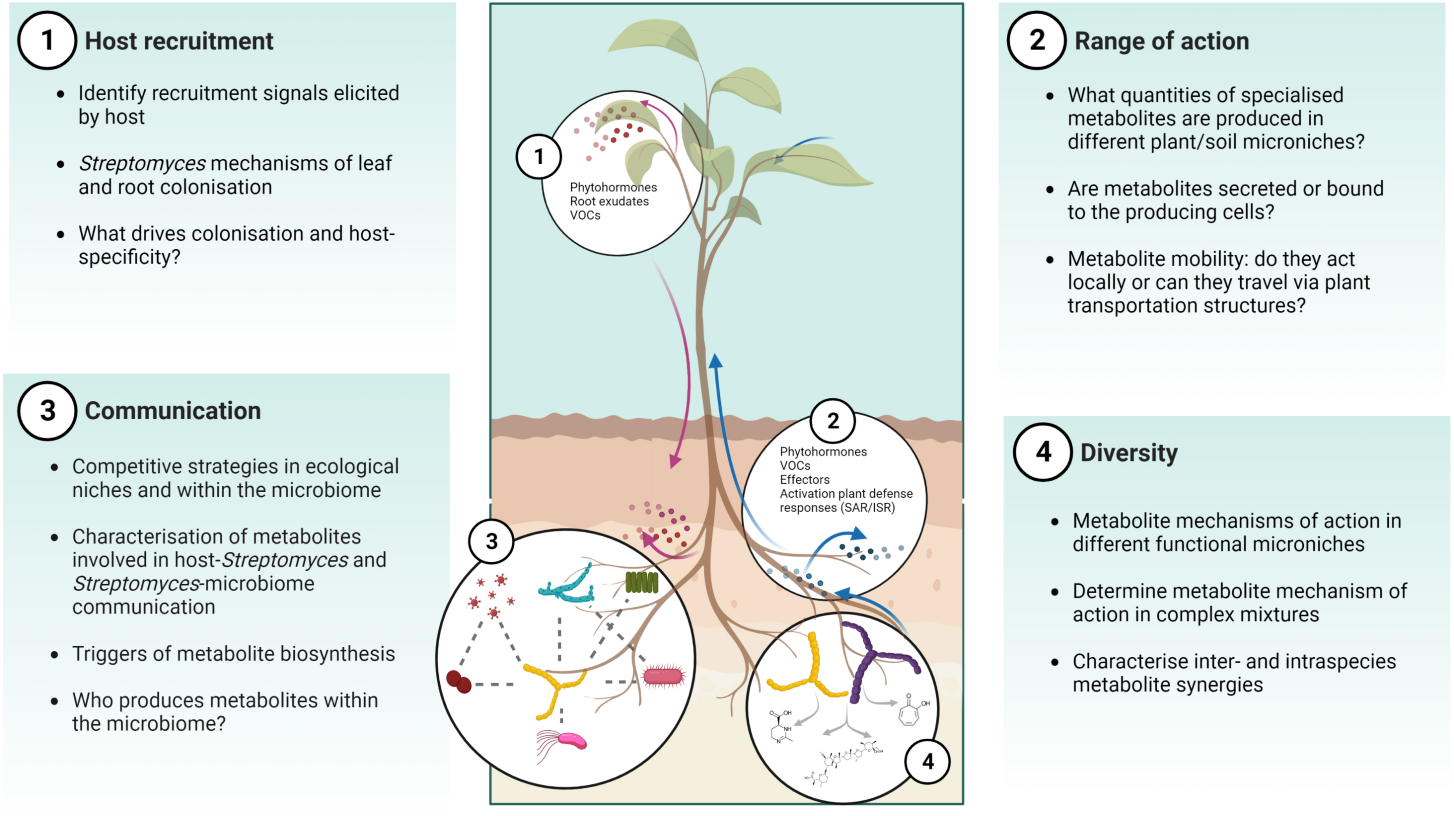
Challenges and opportunities for MBCA specialised metabolite identification and characterisation *in vivo*. There are several gaps in our understanding of the ecological function of *Streptomyces* and their specialised metabolites in the soil-plant environment, including host recruitment, range of action, communication, and diversity. Created with Biorender.

These studies highlight how biosensors incorporated into plant hosts or microbes are important tools to study MBCAs *in planta* or the activity of their secreted specialised metabolites, and how they could be tailored towards real-time spatial or even cellular detection of biological responses. Much like the nascent technique of imaging mass spectrometry, the use of reporter lines *in planta* has the potential to provide information on how MBCA strains behave in the presence of the plant host and the wider microbiome, including many of the unanswered questions posed in **Figure 3**.

## Outlook

The prevalence of *Streptomyces* spp. across soil environments and the relationships they form with plant hosts has attracted interest in their exploitation for crop production. As detailed within this review, they have a long history as MBCAs but their discovery and deployment in agricultural settings has been hindered by ill-suited or narrow-focused *in vitro* bioactivity screens, and the underexplored investigation of their metabolic potential *in planta*. Future work should address questions such as how, what, when, and where *Streptomyces* BGCs are expressed, how do we detect and isolate specialised metabolites, describe their functions in an ecological context, and determine which are most suitable for biocontrol applications (**Figure 3**).

To provide the best outcomes for *Streptomyces* biocontrol agents, future research should prioritise interactions involving *Streptomyces*, the plant host, a target pathogen, and where feasible, assess these dynamic interactions within the context of the host-associated microbiome. Focusing on this tripartite system would also expedite effective MBCA practices, such as their sensitivity to synthetic fungicides (Palazzini et al., 2018; Wachowska et al., 2020). Furthermore, while this review has primarily discussed specialised metabolites originating from *Streptomyces* species, modern analyses of tripartite systems are beginning to highlight how pathogens also alter microbiome structure and the plant-derived metabolome (Seybold et al., 2020; Dove et al., 2022; Jiang et al., 2023).

Despite advances in microbial metabolite identification and characterisation, there are still many challenges to their evaluation *in planta*. New omics tools such as metatranscriptomics and metametabolomics coupled with genetics tools such as gene knockout or gene editing studies provide a pathway to the discovery and dissection of MBCA modes of action *in planta*, of their specialised metabolites and other bioactive compounds, and the interplay between MBCAs and their host and microbiome (**Figure 2**). *In planta* mass spectrometry imaging could be a vital tool to uncover a plethora of novel specialised metabolites and better understand the host-*Streptomyces*-microbiome environment in real-time, and biosensors are promising tools tailored towards studying single metabolites in plant tissues.

Harnessing this new knowledge allows us to identify gaps in MBCA deployment and provides scope to build durability into agricultural systems. Synthetic microbial communities (SynComs), which are small microbial consortia with multiple beneficial microbial traits, could be used to tailor the design of MBCAs (de Souza et al., 2020; Verma et al., 2022). Incorporating the substantial metabolic potential from *Streptomyces* adapted to different environments and microniches could also improve durability. Beyond *Streptomyces*, other species of the phylum Actinobacteria may also offer new functionality with studies isolating rare Actinobacterial strains from root endospheres at higher frequency than rhizospheres or bulk soil (Matsumoto and Takahashi, 2017). Finally, metagenomic tools facilitate the identification of non-culturable *Streptomyces* and other biocontrols, paving the way for the analysis of their specialised metabolite repertoire, their functions, and the signals that elicit their expression *in planta*.

## Author contributions

LD, MG, and LT conceived the review topic and outline. LD, MG, MR, SL, and LT wrote the review. All authors contributed to the article and approved the submitted version.
